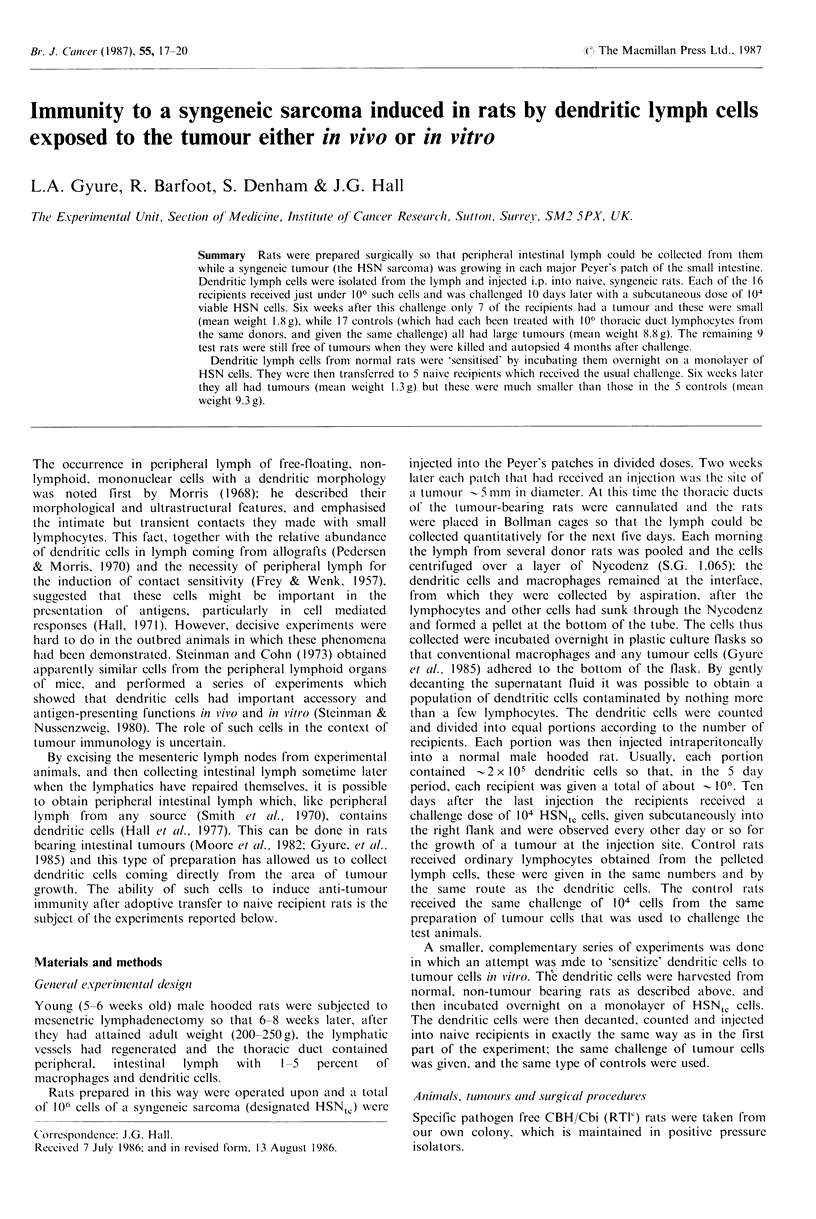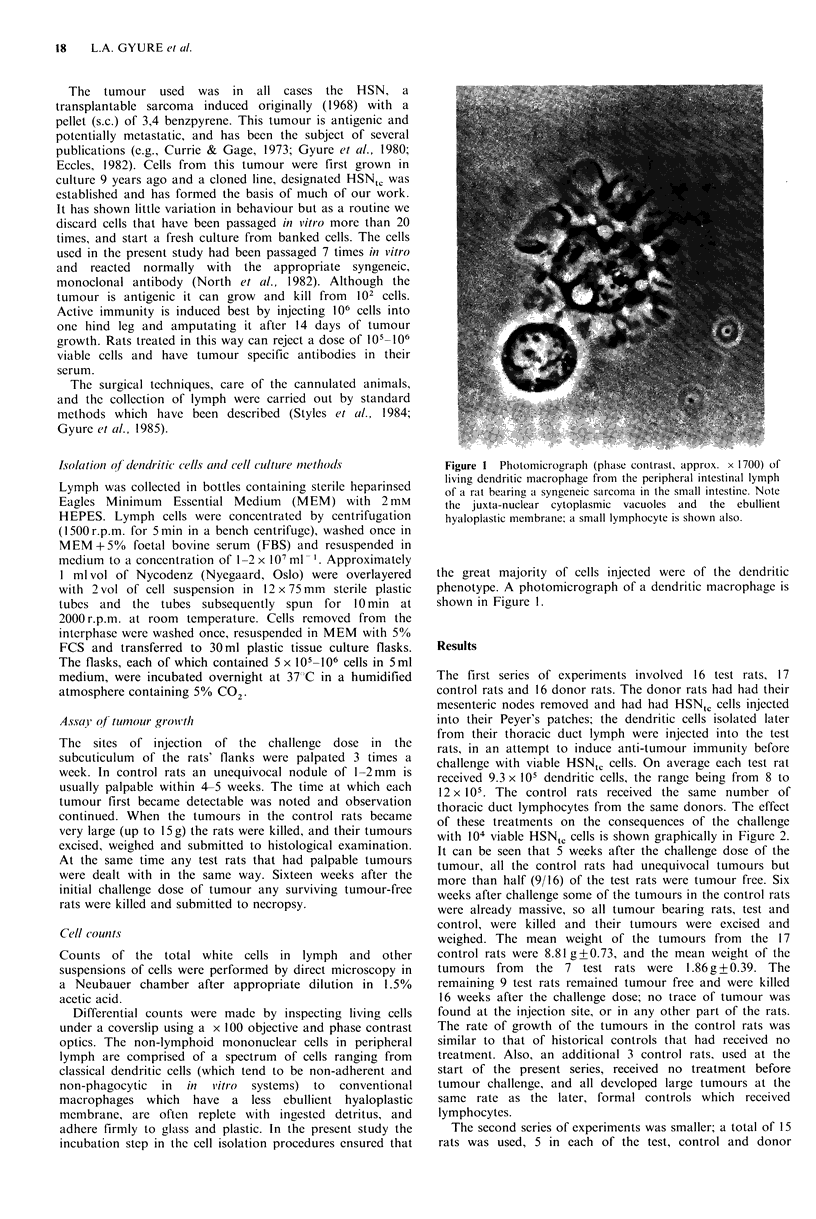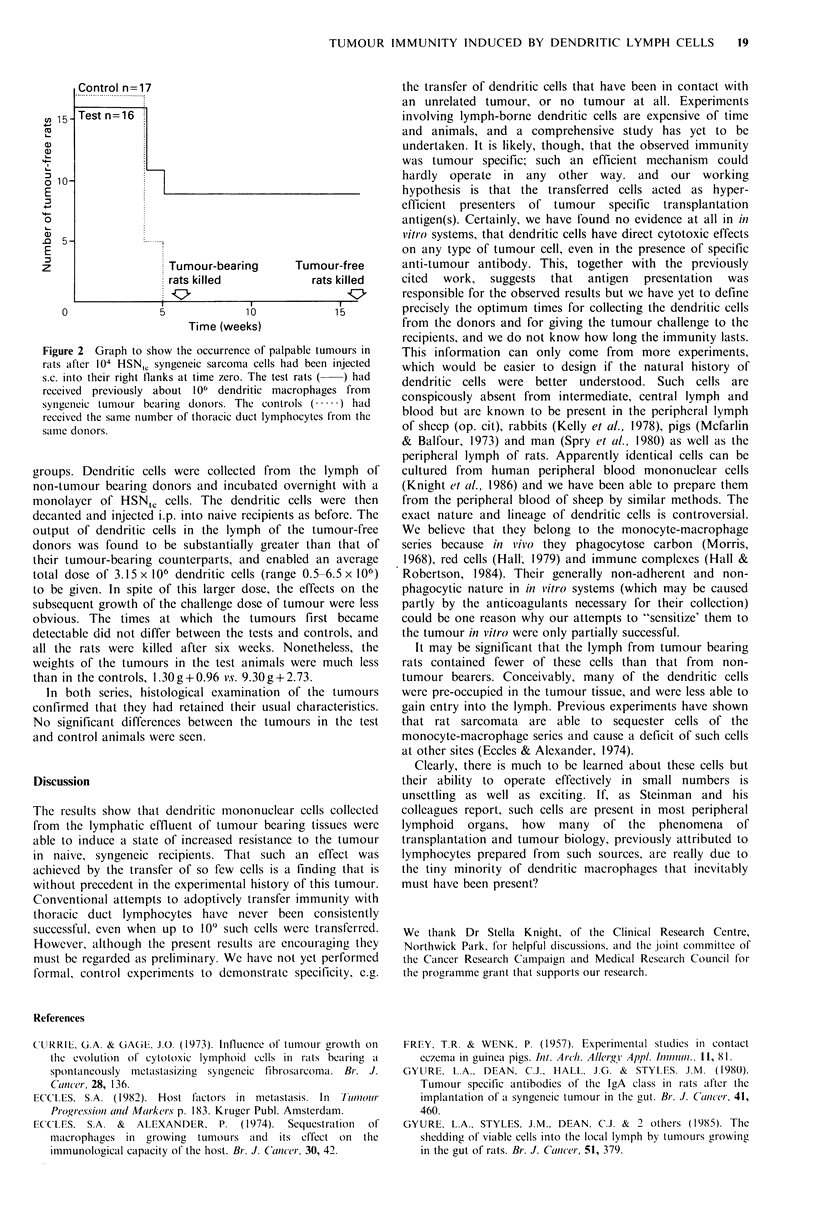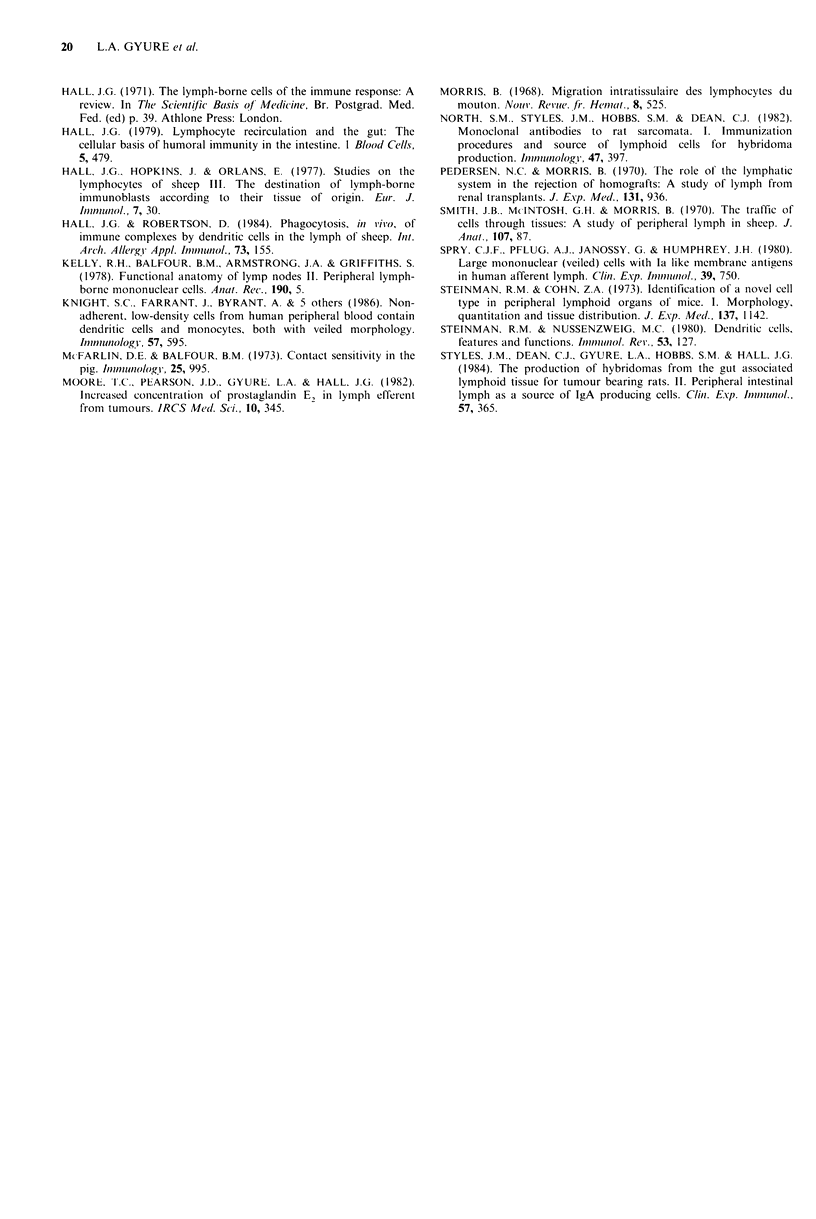# Immunity to a syngeneic sarcoma induced in rats by dendritic lymph cells exposed to the tumour either in vivo or in vitro.

**DOI:** 10.1038/bjc.1987.4

**Published:** 1987-01

**Authors:** L. A. Gyure, R. Barfoot, S. Denham, J. G. Hall

## Abstract

**Images:**


Br. J. Cancer (1987), 55, 17–20 © The Macmillan Press Ltd., 1987

### Immunity to a syngeneic sarcoma induced in rats by dendritic lymph cells exposed to the tumour either *in vivo* or *in vitro*

L.A. Gyure, R. Barfoot, S. Denham & J.G. Hall

*The Experimental Unit, Section of Medicine, Institute of Cancer Research, Sutton, Surrey, SM2 5PX, UK.*

**Summary** Rats were prepared surgically so that peripheral intestinal lymph could be collected from them while a syngeneic tumour (the HSN sarcoma) was growing in each major Peyer's patch of the small intestine. Dendritic lymph cells were isolated from the lymph and injected i.p. into naive, syngeneic rats. Each of the 16 recipients received just under $10^6$ such cells and was challenged 10 days later with a subcutaneous dose of $10^4$ viable HSN cells. Six weeks after this challenge only 7 of the recipients had a tumour and these were small (mean weight 1.8 g), while 17 controls (which had each been treated with $10^6$ thoracic duct lymphocytes from the same donors, and given the same challenge) all had large tumours (mean weight 8.8 g). The remaining 9 test rats were still free of tumours when they were killed and autopsied 4 months after challenge.

Dendritic lymph cells from normal rats were 'sensitised' by incubating them overnight on a monolayer of HSN cells. They were then transferred to 5 naive recipients which received the usual challenge. Six weeks later they all had tumours (mean weight 1.3 g) but these were much smaller than those in the 5 controls (mean weight 9.3 g).

The occurrence in peripheral lymph of free-floating, non-lymphoid, mononuclear cells with a dendritic morphology was noted first by Morris (1968); he described their morphological and ultrastructural features, and emphasised the intimate but transient contacts they made with small lymphocytes. This fact, together with the relative abundance of dendritic cells in lymph coming from allografts (Pedersen & Morris, 1970) and the necessity of peripheral lymph for the induction of contact sensitivity (Frey & Wenk, 1957), suggested that these cells might be important in the presentation of antigens, particularly in cell mediated responses (Hall, 1971). However, decisive experiments were hard to do in the outbred animals in which these phenomena had been demonstrated. Steinman and Cohn (1973) obtained apparently similar cells from the peripheral lymphoid organs of mice, and performed a series of experiments which showed that dendritic cells had important accessory and antigen-presenting functions *in vivo* and *in vitro* (Steinman & Nussenzweig, 1980). The role of such cells in the context of tumour immunology is uncertain.

By excising the mesenteric lymph nodes from experimental animals, and then collecting intestinal lymph sometime later when the lymphatics have repaired themselves, it is possible to obtain peripheral intestinal lymph which, like peripheral lymph from any source (Smith *et al.*, 1970), contains dendritic cells (Hall *et al.*, 1977). This can be done in rats bearing intestinal tumours (Moore *et al.*, 1982; Gyure, *et al.*, 1985) and this type of preparation has allowed us to collect dendritic cells coming directly from the area of tumour growth. The ability of such cells to induce anti-tumour immunity after adoptive transfer to naive recipient rats is the subject of the experiments reported below.

### Materials and methods

### *General experimental design*

Young (5–6 weeks old) male hooded rats were subjected to mesenetric lymphadenectomy so that 6–8 weeks later, after they had attained adult weight (200–250 g), the lymphatic vessels had regenerated and the thoracic duct contained peripheral, intestinal lymph with 1–5 percent of macrophages and dendritic cells.

Rats prepared in this way were operated upon and a total of $10^6$ cells of a syngeneic sarcoma (designated $HSN_{tc}$) were injected into the Peyer's patches in divided doses. Two weeks later each patch that had received an injection was the site of a tumour $\sim$5 mm in diameter. At this time the thoracic ducts of the tumour-bearing rats were cannulated and the rats were placed in Bollman cages so that the lymph could be collected quantitatively for the next five days. Each morning the lymph from several donor rats was pooled and the cells centrifuged over a layer of Nycodenz (S.G. 1.065); the dendritic cells and macrophages remained at the interface, from which they were collected by aspiration, after the lymphocytes and other cells had sunk through the Nycodenz and formed a pellet at the bottom of the tube. The cells thus collected were incubated overnight in plastic culture flasks so that conventional macrophages and any tumour cells (Gyure *et al.*, 1985) adhered to the bottom of the flask. By gently decanting the supernatant fluid it was possible to obtain a population of dendtritic cells contaminated by nothing more than a few lymphocytes. The dendritic cells were counted and divided into equal portions according to the number of recipients. Each portion was then injected intraperitoneally into a normal male hooded rat. Usually, each portion contained $\sim 2\times10^5$ dendritic cells so that, in the 5 day period, each recipient was given a total of about $\sim 10^6$. Ten days after the last injection the recipients received a challenge dose of $10^4$ $HSN_{tc}$ cells, given subcutaneously into the right flank and were observed every other day or so for the growth of a tumour at the injection site. Control rats received ordinary lymphocytes obtained from the pelleted lymph cells, these were given in the same numbers and by the same route as the dendritic cells. The control rats received the same challenge of $10^4$ cells from the same preparation of tumour cells that was used to challenge the test animals.

A smaller, complementary series of experiments was done in which an attempt was mde to 'sensitize' dendritic cells to tumour cells *in vitro*. The dendritic cells were harvested from normal, non-tumour bearing rats as described above, and then incubated overnight on a monolayer of $HSN_{tc}$ cells. The dendritic cells were then decanted, counted and injected into naive recipients in exactly the same way as in the first part of the experiment; the same challenge of tumour cells was given, and the same type of controls were used.

### *Animals, tumours and surgical procedures*

Specific pathogen free CBH/Cbi ($RT1^c$) rats were taken from our own colony, which is maintained in positive pressure isolators.

Correspondence: J.G. Hall.
Received 7 July 1986; and in revised form, 13 August 1986.

The tumour used was in all cases the HSN, a transplantable sarcoma induced originally (1968) with a pellet (s.c.) of 3,4 benzpyrene. This tumour is antigenic and potentially metastatic, and has been the subject of several publications (e.g., Currie & Gage, 1973; Gyure *et al.*, 1980; Eccles, 1982). Cells from this tumour were first grown in culture 9 years ago and a cloned line, designated $HSN_{tc}$ was established and has formed the basis of much of our work. It has shown little variation in behaviour but as a routine we discard cells that have been passaged *in vitro* more than 20 times, and start a fresh culture from banked cells. The cells used in the present study had been passaged 7 times *in vitro* and reacted normally with the appropriate syngeneic, monoclonal antibody (North *et al.*, 1982). Although the tumour is antigenic it can grow and kill from $10^2$ cells. Active immunity is induced best by injecting $10^6$ cells into one hind leg and amputating it after 14 days of tumour growth. Rats treated in this way can reject a dose of $10^5$–$10^6$ viable cells and have tumour specific antibodies in their serum.

The surgical techniques, care of the cannulated animals, and the collection of lymph were carried out by standard methods which have been described (Styles *et al.*, 1984; Gyure *et al.*, 1985).

*Isolation of dendritic cells and cell culture methods*

Lymph was collected in bottles containing sterile heparinsed Eagles Minimum Essential Medium (MEM) with 2 mM HEPES. Lymph cells were concentrated by centrifugation (1500 r.p.m. for 5 min in a bench centrifuge), washed once in MEM + 5% foetal bovine serum (FBS) and resuspended in medium to a concentration of $1$–$2 \times 10^7\,ml^{-1}$. Approximately 1 ml vol of Nycodenz (Nyegaard, Oslo) were overlayered with 2 vol of cell suspension in 12 × 75 mm sterile plastic tubes and the tubes subsequently spun for 10 min at 2000 r.p.m. at room temperature. Cells removed from the interphase were washed once, resuspended in MEM with 5% FCS and transferred to 30 ml plastic tissue culture flasks. The flasks, each of which contained $5 \times 10^5$–$10^6$ cells in 5 ml medium, were incubated overnight at 37°C in a humidified atmosphere containing 5% $CO_2$.

*Assay of tumour growth*

The sites of injection of the challenge dose in the subcuticulum of the rats' flanks were palpated 3 times a week. In control rats an unequivocal nodule of 1–2 mm is usually palpable within 4–5 weeks. The time at which each tumour first became detectable was noted and observation continued. When the tumours in the control rats became very large (up to 15 g) the rats were killed, and their tumours excised, weighed and submitted to histological examination. At the same time any test rats that had palpable tumours were dealt with in the same way. Sixteen weeks after the initial challenge dose of tumour any surviving tumour-free rats were killed and submitted to necropsy.

*Cell counts*

Counts of the total white cells in lymph and other suspensions of cells were performed by direct microscopy in a Neubauer chamber after appropriate dilution in 1.5% acetic acid.

Differential counts were made by inspecting living cells under a coverslip using a × 100 objective and phase contrast optics. The non-lymphoid mononuclear cells in peripheral lymph are comprised of a spectrum of cells ranging from classical dendritic cells (which tend to be non-adherent and non-phagocytic in *in vitro* systems) to conventional macrophages which have a less ebullient hyaloplastic membrane, are often replete with ingested detritus, and adhere firmly to glass and plastic. In the present study the incubation step in the cell isolation procedures ensured that the great majority of cells injected were of the dendritic phenotype. A photomicrograph of a dendritic macrophage is shown in Figure 1.

**Figure 1** Photomicrograph (phase contrast, approx. × 1700) of living dendritic macrophage from the peripheral intestinal lymph of a rat bearing a syngeneic sarcoma in the small intestine. Note the juxta-nuclear cytoplasmic vacuoles and the ebullient hyaloplastic membrane; a small lymphocyte is shown also.

### Results

The first series of experiments involved 16 test rats, 17 control rats and 16 donor rats. The donor rats had had their mesenteric nodes removed and had had $HSN_{tc}$ cells injected into their Peyer's patches; the dendritic cells isolated later from their thoracic duct lymph were injected into the test rats, in an attempt to induce anti-tumour immunity before challenge with viable $HSN_{tc}$ cells. On average each test rat received $9.3 \times 10^5$ dendritic cells, the range being from 8 to $12 \times 10^5$. The control rats received the same number of thoracic duct lymphocytes from the same donors. The effect of these treatments on the consequences of the challenge with $10^4$ viable $HSN_{tc}$ cells is shown graphically in Figure 2. It can be seen that 5 weeks after the challenge dose of the tumour, all the control rats had unequivocal tumours but more than half (9/16) of the test rats were tumour free. Six weeks after challenge some of the tumours in the control rats were already massive, so all tumour bearing rats, test and control, were killed and their tumours were excised and weighed. The mean weight of the tumours from the 17 control rats were $8.81\,g \pm 0.73$, and the mean weight of the tumours from the 7 test rats were $1.86\,g \pm 0.39$. The remaining 9 test rats remained tumour free and were killed 16 weeks after the challenge dose; no trace of tumour was found at the injection site, or in any other part of the rats. The rate of growth of the tumours in the control rats was similar to that of historical controls that had received no treatment. Also, an additional 3 control rats, used at the start of the present series, received no treatment before tumour challenge, and all developed large tumours at the same rate as the later, formal controls which received lymphocytes.

The second series of experiments was smaller; a total of 15 rats was used, 5 in each of the test, control and donor

**Figure 2** Graph to show the occurrence of palpable tumours in rats after $10^4$ $HSN_{tc}$ syngeneic sarcoma cells had been injected s.c. into their right flanks at time zero. The test rats (——) had received previously about $10^6$ dendritic macrophages from syngeneic tumour bearing donors. The controls (·····) had received the same number of thoracic duct lymphocytes from the same donors.

groups. Dendritic cells were collected from the lymph of non-tumour bearing donors and incubated overnight with a monolayer of $HSN_{tc}$ cells. The dendritic cells were then decanted and injected i.p. into naive recipients as before. The output of dendritic cells in the lymph of the tumour-free donors was found to be substantially greater than that of their tumour-bearing counterparts, and enabled an average total dose of $3.15 \times 10^6$ dendritic cells (range 0.5–$6.5 \times 10^6$) to be given. In spite of this larger dose, the effects on the subsequent growth of the challenge dose of tumour were less obvious. The times at which the tumours first became detectable did not differ between the tests and controls, and all the rats were killed after six weeks. Nonetheless, the weights of the tumours in the test animals were much less than in the controls, 1.30 g + 0.96 *vs.* 9.30 g + 2.73.

In both series, histological examination of the tumours confirmed that they had retained their usual characteristics. No significant differences between the tumours in the test and control animals were seen.

### Discussion

The results show that dendritic mononuclear cells collected from the lymphatic effluent of tumour bearing tissues were able to induce a state of increased resistance to the tumour in naive, syngeneic recipients. That such an effect was achieved by the transfer of so few cells is a finding that is without precedent in the experimental history of this tumour. Conventional attempts to adoptively transfer immunity with thoracic duct lymphocytes have never been consistently successful, even when up to $10^9$ such cells were transferred. However, although the present results are encouraging they must be regarded as preliminary. We have not yet performed formal, control experiments to demonstrate specificity, e.g. the transfer of dendritic cells that have been in contact with an unrelated tumour, or no tumour at all. Experiments involving lymph-borne dendritic cells are expensive of time and animals, and a comprehensive study has yet to be undertaken. It is likely, though, that the observed immunity was tumour specific; such an efficient mechanism could hardly operate in any other way. and our working hypothesis is that the transferred cells acted as hyper-efficient presenters of tumour specific transplantation antigen(s). Certainly, we have found no evidence at all in *in vitro* systems, that dendritic cells have direct cytotoxic effects on any type of tumour cell, even in the presence of specific anti-tumour antibody. This, together with the previously cited work, suggests that antigen presentation was responsible for the observed results but we have yet to define precisely the optimum times for collecting the dendritic cells from the donors and for giving the tumour challenge to the recipients, and we do not know how long the immunity lasts. This information can only come from more experiments, which would be easier to design if the natural history of dendritic cells were better understood. Such cells are conspicously absent from intermediate, central lymph and blood but are known to be present in the peripheral lymph of sheep (op. cit), rabbits (Kelly *et al.*, 1978), pigs (Mcfarlin & Balfour, 1973) and man (Spry *et al.*, 1980) as well as the peripheral lymph of rats. Apparently identical cells can be cultured from human peripheral blood mononuclear cells (Knight *et al.*, 1986) and we have been able to prepare them from the peripheral blood of sheep by similar methods. The exact nature and lineage of dendritic cells is controversial. We believe that they belong to the monocyte-macrophage series because *in vivo* they phagocytose carbon (Morris, 1968), red cells (Hall, 1979) and immune complexes (Hall & Robertson, 1984). Their generally non-adherent and non-phagocytic nature in *in vitro* systems (which may be caused partly by the anticoagulants necessary for their collection) could be one reason why our attempts to "sensitize' them to the tumour *in vitro* were only partially successful.

It may be significant that the lymph from tumour bearing rats contained fewer of these cells than that from non-tumour bearers. Conceivably, many of the dendritic cells were pre-occupied in the tumour tissue, and were less able to gain entry into the lymph. Previous experiments have shown that rat sarcomata are able to sequester cells of the monocyte-macrophage series and cause a deficit of such cells at other sites (Eccles & Alexander, 1974).

Clearly, there is much to be learned about these cells but their ability to operate effectively in small numbers is unsettling as well as exciting. If, as Steinman and his colleagues report, such cells are present in most peripheral lymphoid organs, how many of the phenomena of transplantation and tumour biology, previously attributed to lymphocytes prepared from such sources, are really due to the tiny minority of dendritic macrophages that inevitably must have been present?

We thank Dr Stella Knight, of the Clinical Research Centre, Northwick Park, for helpful discussions, and the joint committee of the Cancer Research Campaign and Medical Research Council for the programme grant that supports our research.

### References

CURRIE, G.A. & GAGE, J.O. (1973). Influence of tumour growth on the evolution of cytotoxic lymphoid cells in rats bearing a spontaneously metastasizing syngeneic fibrosarcoma. *Br. J. Cancer*, **28,** 136.

ECCLES, S.A. (1982). Host factors in metastasis. In *Tumour Progression and Markers* p. 183. Kruger Publ. Amsterdam.

ECCLES, S.A. & ALEXANDER, P. (1974). Sequestration of macrophages in growing tumours and its effect on the immunological capacity of the host. *Br. J. Cancer*, **30,** 42.

FREY, T.R. & WENK, P. (1957). Experimental studies in contact eczema in guinea pigs. *Int. Arch. Allergy Appl. Immun.*, **11,** 81.

GYURE, L.A., DEAN, C.J., HALL, J.G. & STYLES, J.M. (1980). Tumour specific antibodies of the IgA class in rats after the implantation of a syngeneic tumour in the gut. *Br. J. Cancer*, **41,** 460.

GYURE, L.A., STYLES, J.M., DEAN, C.J. & 2 others (1985). The shedding of viable cells into the local lymph by tumours growing in the gut of rats. *Br. J. Cancer*, **51,** 379.

HALL, J.G. (1971). The lymph-borne cells of the immune response: A review. In *The Scientific Basis of Medicine*, Br. Postgrad. Med. Fed. (ed) p. 39. Athlone Press: London.

HALL, J.G. (1979). Lymphocyte recirculation and the gut: The cellular basis of humoral immunity in the intestine. I *Blood Cells*, **5**, 479.

HALL, J.G., HOPKINS, J. & ORLANS, E. (1977). Studies on the lymphocytes of sheep III. The destination of lymph-borne immunoblasts according to their tissue of origin. *Eur. J. Immunol.*, **7**, 30.

HALL, J.G. & ROBERTSON, D. (1984). Phagocytosis, *in vivo*, of immune complexes by dendritic cells in the lymph of sheep. *Int. Arch. Allergy Appl. Immunol.*, **73**, 155.

KELLY, R.H., BALFOUR, B.M., ARMSTRONG, J.A. & GRIFFITHS, S. (1978). Functional anatomy of lymp nodes II. Peripheral lymph-borne mononuclear cells. *Anat. Rec.*, **190**, 5.

KNIGHT, S.C., FARRANT, J., BYRANT, A. & 5 others (1986). Non-adherent, low-density cells from human peripheral blood contain dendritic cells and monocytes, both with veiled morphology. *Immunology*, **57**, 595.

McFARLIN, D.E. & BALFOUR, B.M. (1973). Contact sensitivity in the pig. *Immunology*, **25**, 995.

MOORE, T.C., PEARSON, J.D., GYURE, L.A. & HALL, J.G. (1982). Increased concentration of prostaglandin $E_2$ in lymph efferent from tumours. *IRCS Med. Sci.*, **10**, 345.

MORRIS, B. (1968). Migration intratissulaire des lymphocytes du mouton. *Nouv. Revue. fr. Hemat.*, **8**, 525.

NORTH, S.M., STYLES, J.M., HOBBS, S.M. & DEAN, C.J. (1982). Monoclonal antibodies to rat sarcomata. I. Immunization procedures and source of lymphoid cells for hybridoma production. *Immunology*, **47**, 397.

PEDERSEN, N.C. & MORRIS, B. (1970). The role of the lymphatic system in the rejection of homografts: A study of lymph from renal transplants. *J. Exp. Med.*, **131**, 936.

SMITH, J.B., McINTOSH, G.H. & MORRIS, B. (1970). The traffic of cells through tissues: A study of peripheral lymph in sheep. *J. Anat.*, **107**, 87.

SPRY, C.J.F., PFLUG, A.J., JANOSSY, G. & HUMPHREY, J.H. (1980). Large mononuclear (veiled) cells with Ia like membrane antigens in human afferent lymph. *Clin. Exp. Immunol.*, **39**, 750.

STEINMAN, R.M. & COHN, Z.A. (1973). Identification of a novel cell type in peripheral lymphoid organs of mice. I. Morphology, quantitation and tissue distribution. *J. Exp. Med.*, **137**, 1142.

STEINMAN, R.M. & NUSSENZWEIG, M.C. (1980). Dendritic cells, features and functions. *Immunol. Rev.*, **53**, 127.

STYLES, J.M., DEAN, C.J., GYURE, L.A., HOBBS, S.M. & HALL, J.G. (1984). The production of hybridomas from the gut associated lymphoid tissue for tumour bearing rats. II. Peripheral intestinal lymph as a source of IgA producing cells. *Clin. Exp. Immunol.*, **57**, 365.